# Two critical positions in zinc finger domains are heavily mutated in three human cancer types

**DOI:** 10.1371/journal.pcbi.1006290

**Published:** 2018-06-28

**Authors:** Daniel Munro, Dario Ghersi, Mona Singh

**Affiliations:** 1 Lewis-Sigler Institute for Integrative Genomics, Princeton University, Princeton, NJ, USA; 2 School of Interdisciplinary Informatics, University of Nebraska at Omaha, Omaha, NE, USA; 3 Department of Computer Science, Princeton University, Princeton, NJ, USA; Center for Cancer Research, UNITED KINGDOM

## Abstract

A major goal of cancer genomics is to identify somatic mutations that play a role in tumor initiation or progression. Somatic mutations within transcription factors are of particular interest, as gene expression dysregulation is widespread in cancers. The substantial gene expression variation evident across tumors suggests that numerous regulatory factors are likely to be involved and that somatic mutations within them may not occur at high frequencies across patient cohorts, thereby complicating efforts to uncover which ones are cancer-relevant. Here we analyze somatic mutations within the largest family of human transcription factors, namely those that bind DNA via Cys2His2 zinc finger domains. Specifically, to hone in on important mutations within these genes, we aggregated somatic mutations across all of them by their positions within Cys2His2 zinc finger domains. Remarkably, we found that for three classes of cancers profiled by The Cancer Genome Atlas (TCGA)—Uterine Corpus Endometrial Carcinoma, Colon and Rectal Adenocarcinomas, and Skin Cutaneous Melanoma—two specific, functionally important positions within zinc finger domains are mutated significantly more often than expected by chance, with alterations in 18%, 10% and 43% of tumors, respectively. Numerous zinc finger genes are affected, with those containing Krüppel-associated box (KRAB) repressor domains preferentially targeted by these mutations. Further, the genes with these mutations also have high overall missense mutation rates, are expressed at levels comparable to those of known cancer genes, and together have biological process annotations that are consistent with roles in cancers. Altogether, we introduce evidence broadly implicating mutations within a diverse set of zinc finger proteins as relevant for cancer, and propose that they contribute to the widespread transcriptional dysregulation observed in cancer cells.

## Author summary

Recent large-scale cancer genomics initiatives have revealed that tumor cells typically have numerous genetic alterations, only a small subset of which play a functional role in cancer. Thus, discovering cancer-relevant mutations from tumor sequencing data is a major goal of cancer genomics. Here, we aimed to uncover functional mutations occurring in transcription factors, as gene expression dysregulation is frequently observed in human cancers. In particular, we analyzed Cys2His2 zinc finger genes, as they comprise the largest family of transcription factors in human. We aggregated somatic mutations within these genes by their positions within zinc finger domains, and found that two specific positions within zinc finger domains are recurrently mutated in three cancer types—Uterine Corpus Endometrial Carcinoma, Colon and Rectal Adenocarcinomas, and Skin Cutaneous Melanoma. The two heavily altered positions are known to influence whether and how the domains bind DNA, and thus mutations within them can substantially alter gene function. Further, these mutations, observed across a wide range of transcription factors, converge on at least two processes—chromatin remodeling and dysregulation of retroelements—that are increasingly being linked to human cancers. More generally, we propose that these uncovered mutations contribute to the widespread transcriptional dysregulation commonly observed in cancer cells.

## Introduction

Recent cancer genomics efforts have sequenced the tumors of hundreds of individuals across tens of different cancer types [1, 2]. Analyses of these genomes have confirmed that cancer is a highly heterogeneous disease, with tumors of the same cancer type exhibiting numerous distinct somatic alterations. Uncovering which of these mutations play a functional role in oncogenesis or tumor progression is critical for furthering our understanding of cancers and for uncovering new therapeutic targets. While approaches based on identifying genes mutated across cancer samples more often than expected by chance have yielded both well-known and newly predicted cancer genes [3], the level of heterogeneity observed in cancers suggests that there are many genes that are mutated across smaller subsets of individuals but nevertheless play key roles in cancer progression.

Previously, analysis of protein structures revealed that many well-known cancer genes are enriched in mutations that affect protein stability or participation in interactions with nucleic acids, small molecules and peptides [4–6]. Thus, these types of somatic mutations are promising as cancer-relevant candidates, even if they occur infrequently across patient cohorts. Mutations of residues that affect the stability and specificity of DNA-binding domains are particularly noteworthy, as they can contribute to gene expression dysregulation, a widespread but highly varied phenomenon across tumors [7]. Indeed, mutations within DNA-binding proteins such as p53 [8], ARID1A [9], and GATA3 [10] are prevalent in cancers. However, the heterogeneity of gene expression profiles across cancers suggests that numerous less frequently mutated transcription factors may also be relevant for cancer.

To expand our knowledge of DNA-binding proteins that may play a role in human cancers, we analyzed somatic mutations found in Cys2His2 zinc finger (ZF) domains. ZF domains are major determinants of human regulatory networks, as they are contained in nearly half of human transcription factors [11]. Despite the frequency of ZF domains, the functions of most human ZF proteins remain largely mysterious. Nevertheless, individual ZF proteins have been implicated in a range of important biological processes, including apoptosis, cell differentiation, cell proliferation, and chromosomal organization [12]. Further, the largest subclass of human ZF proteins additionally contain Krüppel-associated box (KRAB) domains, and most of these proteins are thought to mediate transcriptional repression via interactions with chromatin-remodeling factors [13, 14] and many of them have been found to bind and repress retroelements [15–18]; these ZFs may be of particular interest as changes in chromatin and altered expression of transposable elements are both observed in cancers [19, 20].

The structurally and functionally important positions within ZF domains are well known [21], and this knowledge provides a unifying framework within which to evaluate somatic mutations—even if relatively infrequent—that are found across a heterogeneous set of ZF genes. In particular, we reasoned that since specific positions within the ZF domain are associated with distinct functional roles, such as stabilizing structure or influencing the interaction with DNA, individual positions may be under differing selective pressure in human cancers. Therefore, we aggregated somatic mutations across multiple proteins by the position they fell into within ZF domains, a method that has been previously proposed to identify mutation hotspots within domains [22]. We then assessed whether positions were mutated more often than expected when controlling for mutation rates within genes and tumor samples as well as for cancer-specific mutational signatures.

Our main finding is that two specific positions within ZF domains are mutated in three cancer types—Uterine Corpus Endometrial Carcinoma (UCEC), Colon and Rectal Adenocarcinomas (COAD/READ), and Skin Cutaneous Melanoma (SKCM)—significantly more often than expected. These two heavily altered positions are known via previous structural studies to influence DNA-binding activity [21, 23]. The uncovered mutations are found in numerous genes, and are enriched in those containing Krüppel-associated box (KRAB) repressor domains. We also demonstrate that genes affected by these mutations have high overall missense mutation rates, are expressed at levels comparable to those of known driver genes, and in aggregate have biological process annotations that are consistent with roles in cancers. Overall, our work implicates a diverse set of ZF proteins as functionally relevant for cancer, and we propose that mutations within these proteins contribute to the pervasive transcriptional dysregulation observed in cancer cells.

## Results

**Fig 1:**
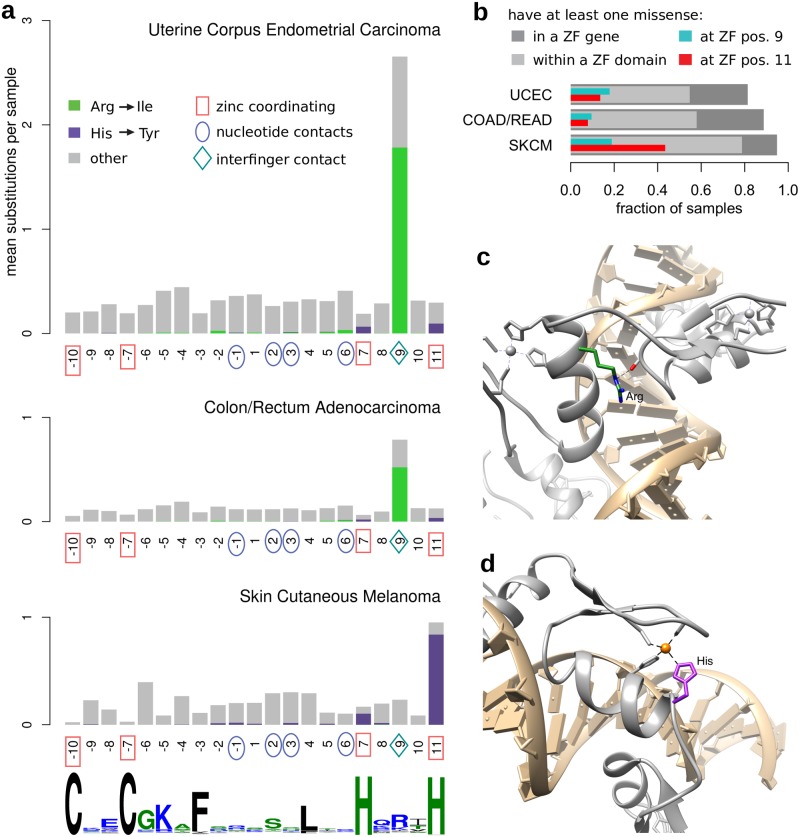
Cys2His2 zinc finger domains show specific mutation patterns in human cancers. **(a)** For each cancer type, somatic mutations occurring within all human Cys2His2 ZF domains were aggregated by amino acid position within the domains, and the average number of mutations per tumor sample is shown. Positions within the domain are numbered relative to the start of the *α*-helix that binds DNA, as is standard for ZF domains [21]. Prominent peaks are evident in position 9 in Uterine Endometrial Corpus Carcinoma and Colorectal Adenocarcinoma, and position 11 in Skin Cutaneous Melanoma, with arginine to isoleucine mutations shown in green, and histidine to tyrosine mutations shown in purple. The structural roles of ZF amino acid positions involved in zinc coordination, nucleotide contact, and inter-finger contact are indicated. A sequence logo of all human Cys2His2 ZF domains is shown below for reference. **(b)** For each cancer type, bars represent the fraction of total patients who have at least one missense mutation within a ZF gene, within a ZF domain, at position 9 of a ZF, and at position 11 of a ZF. **(c)** The arginine at position 9 (colored in green)—frequently mutated into an isoleucine in uterine and colorectal cancers—has the potential to form a hydrogen-bond with the adjacent ZF. **(d)** The histidine at position 11 (colored in purple)—typically mutated into a tyrosine in melanoma—is required for coordinating the zinc ion. (PDB code: 2gli [24]).

We identified 642 human genes containing 5483 “classic” Cys2His2 ZF domains, and determined the frequency with which each of the 21 positions in this domain was mutated across patient cohorts of 32 different cancer types (see Methods). Strikingly, we found that specific positions within ZF domains were recurrently mutated in three classes of cancers: Uterine Corpus Endometrial Carcinoma (UCEC), Colon and Rectal Adenocarcinomas (COAD/READ) and Skin Cutaneous Melanoma (SKCM) (Fig 1a). Position 9 (p9, numbered relative to the start of the *α*-helix that binds DNA) is mutated frequently in the UCEC and COAD/READ cohorts, largely in the form of an arginine to isoleucine mutation (R9I). Position 11 (p11) is mutated frequently in the SKCM cohort, almost exclusively as a histidine to tyrosine mutation (H11Y). These mutations affect a considerable fraction of tumors, with missense mutations at p9 found in 97 of 543 UCEC and 57 of 594 COAD/READ tumors, and missense mutations at p11 found in 204 of 470 SKCM tumors (Fig 1b). Further, a diverse set of ZF genes harbor these mutations, with p9 missense mutations in 367 and 228 genes in UCEC and COAD/READ, respectively, and p11 missense mutations in 199 genes in SKCM.

Positions 9 and 11 both play critical structural roles in ZF domains (Fig 1c and 1d). The histidine in p11 is one of four residues that coordinate zinc, and thereby is essential for stabilizing the domain; while this position can accomodate a cysteine and still coordinate zinc, substitutions of other residues lead to a loss of domain function [21, 25]. ZF proteins typically contain several ZF domains, with closely linked, adjacent ZF domains working together to bind DNA. Arginine in p9 stabilizes the docking of adjacent ZF domains via a contact with the backbone carbonyl or side chain at position -2 of the adjacent C-terminal ZF, and residues in p9 influence the orientation between these domains, an important factor in DNA recognition [21, 23]. Consistent with this functional role within arrays of ZF domains, ZF domains with R9I mutations have adjacent C-terminal ZF domains more often than expected, whereas this is not the case for ZF domains with H11Y mutations (S1 Fig). Given the key roles that p9 and p11 play in ZF structure and function, mutations at these positions are likely to alter or disrupt the DNA-binding properties of the proteins in which they are found.

### Highly enriched number of mutations in zinc finger positions 9 and 11

**Fig 2:**
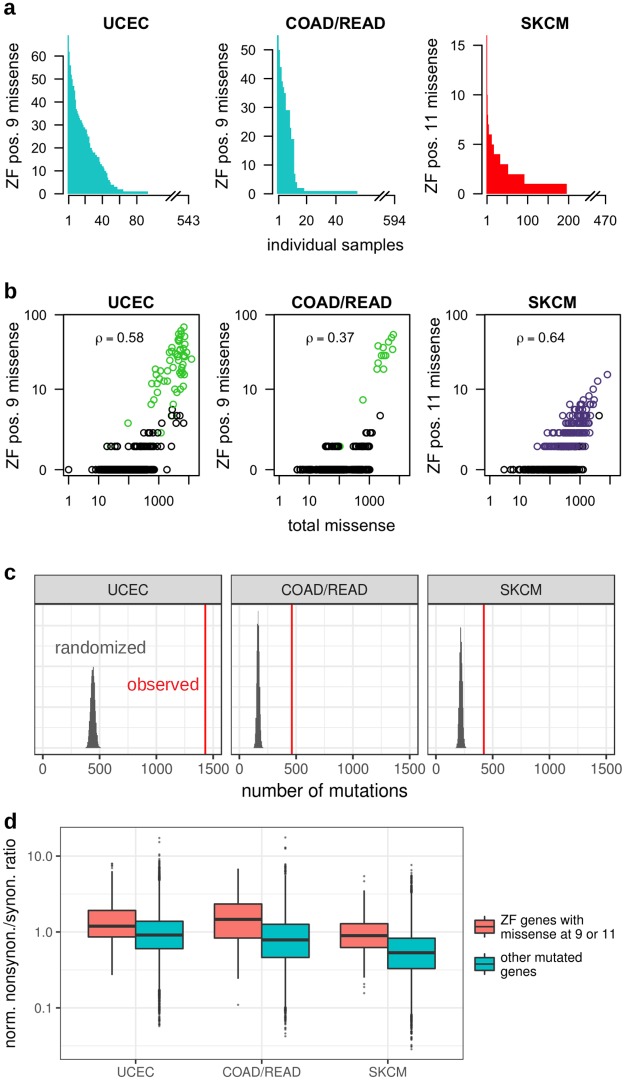
Widespread and enriched mutations across patient samples. **(a)** Per-individual numbers, sorted from high to low, of somatic mutations falling in ZF position 9 in UCEC and COAD/READ, and in ZF position 11 in SKCM. **(b)** For each UCEC and COAD/READ tumor sample with a mutation in position 9 and each SKCM tumor sample with a mutation in position 11, the number of missense mutations at the specified ZF position is plotted against that patient’s total number of missense mutations. The Spearman’s rank correlation coefficient between these values is given in each panel. Samples with at least one R9I/H11Y mutation are shown in green/purple, respectively. **(c)** The histograms display the number of missense mutations in the positions of interest obtained in 10,000 trinucleotide context-preserving randomizations, while the red line shows the actual observed number. This permutation test reveals that the excess of missense mutations in positions 9 and 11 is statistically significant. **(d)** Box plots of the per-gene ratios of normalized nonsynonymous to synonymous mutation counts. ZF genes with at least one missense mutation at position 9 (UCEC, COAD/READ) or 11 (SKCM) tend to have higher ratios than other mutated genes (one-sided Mann-Whitney test *p*-values 2.4e-23, 1.1e-27, and 8.8e-28, respectively).

Having observed recurrent mutations at two functionally crucial positions within ZF domains, we next sought to uncover whether these positions are altered more often than expected by chance. The numbers of p9 and p11 mutations vary substantially across individual tumors (Fig 2a). Further, since the number of these mutations observed in each tumor is positively correlated with the total number of missense mutations (Fig 2b), consideration of per-individual mutation rates is necessary to ascertain the significance of these two mutational peaks. Thus, we obtained a background model by performing trinucleotide context-preserving permutations of mutations within each of the 642 ZF genes (see Methods). The total actual number of missense mutations at p9 is 3.2 and 2.8 times the expected count for UCEC and COAD/READ, respectively, and at p11 is 1.9 times the expected count for SKCM (*p* < 0.0001, Fig 2c).

UCEC and COAD/READ tumors with mutations within the exonuclease domain of DNA polymerase *ϵ* (*POLE*) are ultramutated [26, 27]. To exclude the possibility that mutations at p9 arise solely from the ultramutator phenotype, we repeated the trinucleotide context-sensitive permutation analysis on the 1,074 UCEC and COAD/READ samples without missense mutations in the exonuclease domain of *POLE*. Even within this subset of individuals with lower overall mutational load, there are still (respectively) 2.1 and 1.5 times as many p9 missense mutations as expected (*p* < 0.0001). Notably, for all three cancers, other positions in the ZF domain have missense mutation frequencies that are roughly as expected using this permutation procedure (S2 Fig), indicating that after accounting for per-gene and per-individual mutational contexts, p9 and p11 are specifically enriched for mutation accumulation across these cancer cohorts.

### Zinc finger mutations may arise from cancer type-specific mutational signatures but occur more often than expected

Because different cancers exhibit distinct mutational signatures, we next considered ZF mutations in light of these cancer-specific mutational biases. Missense mutations at p9 cause arginine to isoleucine substitutions 67% and 66% of the time for UCEC and COAD/READ, respectively. Since arginine can only mutate to isoleucine via an AGA→ATA transversion, we compared the number of p9 AGA codons that are mutated in all ZF genes per tumor to what is expected based upon per-tumor rates of AGA trinucleotide mutations across all coding regions (see Methods). Remarkably, the total numbers of p9 AGA mutations across cohorts of UCEC and COAD/READ individuals are 9.4 and 9.9 times higher than expected (both *p*-values < 1e-10, Poisson binomial test).

UCEC and COAD/READ tumors with the *POLE* ultramutator phenotype show a significant increase in the G:C→T:A transversion rate, particularly when flanked by an A:T base pair [26–29]. Indeed, of the 55 UCEC and 15 COAD/READ tumors with R9I mutations, 57 have missense mutations within the exonuclease domain of *POLE*, and an additional 3 have missense mutations elsewhere in *POLE*. When restricting our analysis to the 63 UCEC and COAD/READ samples with missense mutations in the exonuclease domain of *POLE*, the numbers of p9 AGA mutations remain enriched as compared to what is expected based upon the per-exome background rates of AGA mutations in these cancers (10.8 and 12.1 times higher, *p* = 1.0e-12 and *p* = 3.6e-13, Poisson binomial test). Thus, while these arginine to isoleucine mutations are consistent with the ultramutator phenotype, they accumulate at p9 of ZF domains at an unexpectedly high rate.

In SKCM, the H11Y mutations involve a Cytosine to Thymine mutation in the first position of the histidine codon, and occur when the Cytosine is preceded by a pyrimidine; the mutational patterns seen with ultraviolet light exposure and in melanoma consist of frequent CC→CT and TC→TT mutations [30]. However, ZF H11Y mutations occur 13.5 times more frequently than expected (*p* = 4.4e-11, Poisson binomial test) when considering the per-exome rates of CC→CT and TC→TT mutations. Thus, as with the R9I mutations and the ultramutator phenotype in UCEC and COAD/READ, the H11Y mutations are in concordance with the mutational profile characteristic of skin cancers but occur significantly more frequently than expected.

### Zinc finger genes with position 9 and position 11 mutations tend to have higher missense mutation rates than expected

Having demonstrated that p9 and p11 mutations are enriched in ZF domains, we next examined the overall missense mutation rates of the genes within which they are found, as cancer-relevant genes are more likely to be recurrently mutated. Genes with a missense mutation at p9 (UCEC and COAD/READ) or p11 (SKCM) harbor missense mutations anywhere in their sequences in a moderate but clinically relevant range of individuals (each gene is mutated in, on average, 3.7%, 1.6% and 3.2% of UCEC, COAD/READ and SKCM tumors respectively). We next calculated for these genes the rate of missense mutations per coding sequence base, averaged across all tumor samples for each gene, while excluding the missense mutations at these ZF positions. Missense mutation rates are significantly higher in these genes than in other genes (median values for the former are 16.8%, 33.5% and 19.0% higher than those of the latter, even though rates for the latter contain all missense mutations, with *p*-values 8.5e-15, 2.3e-14, and 1.6e-5 for UCEC, COAD/READ, and SKCM, respectively, Mann-Whitney U test).

We next excluded the possibility that these higher missense mutation rates reflect higher overall mutation rates in the genomic regions of these genes. For each cancer type, we divided the number of missense mutations in each gene by the total number of mutations that would lead to nonsynonymous changes in that gene, and likewise with synonymous mutations (see Methods). While the overall distribution of the ratios of these two values are affected by the trinucleotide mutation profiles of each cancer type, they are comparable across genes within each cancer type since they account for both gene length and codon composition. The ratios for ZF genes with at least one missense mutation at p9 (UCEC, COAD/READ) or p11 (SKCM) are higher than the ratios for all other genes with missense and synonymous mutations (one-sided Mann-Whitney *p*-values 2.4e-23, 1.1e-27, and 8.8e-28, respectively) (Fig 2d). We conclude that the set of ZF genes with mutations at p9 and p11 generally have higher missense mutation rates than expected based upon their synonymous mutation rates, thereby ruling out the explanation that these genes or their genomic locations are simply more mutable, and instead lending support to the relevance of these genes to cancers.

### Mutated zinc finger genes are expressed at levels comparable to other genes relevant for these cancers

**Fig 3:**
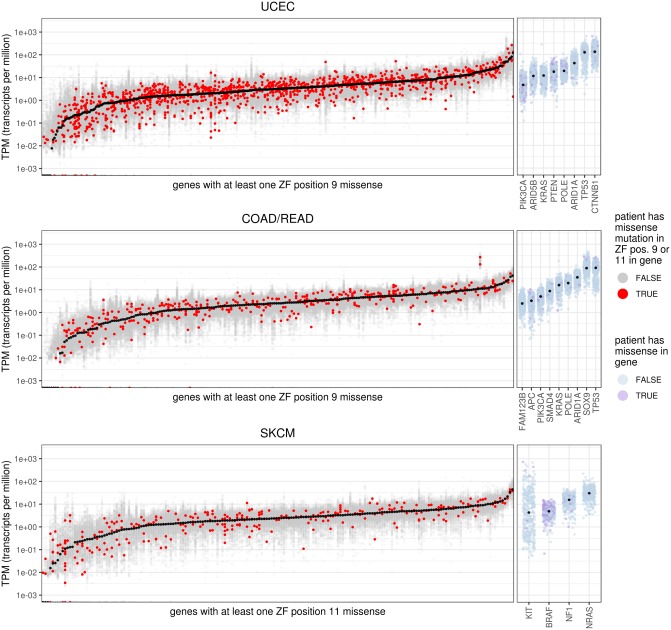
Mutated zinc finger proteins are expressed at levels comparable to other cancer genes. For each cancer type, the per-tumor expression levels in transcripts per million (TPM) are shown for genes with at least one ZF position 9 (UCEC and COAD/READ) or 11 (SKCM) missense mutation. Genes are ordered by their median TPM level across individuals (shown in black). Expression levels for genes in individuals where that gene has a mutation in the position of interest are shown in red, whereas all other levels are shown in grey. For comparison, TPM values for some noteworthy genes previously proposed as being functionally important in the corresponding cancers are shown [28, 29, 31].

To confirm that the uncovered mutations are affecting genes that are expressed, we analyzed RNA-seq data from TCGA for all ZF genes containing a missense mutation in at least one individual at p9 in COAD/READ and UCEC and p11 in SKCM (Fig 3). For comparison, we also extracted gene expression values for genes that are identified in the abstracts of TCGA studies as significantly mutated in these cancers [28, 29, 31]. The expression levels of the mutated ZF genes overlap considerably with those of the previously implicated cancer genes. Further, for each ZF gene, the expression levels in samples where they are mutated in p9 (UCEC and COAD/READ) or p11 (SKCM) are similar to those where they are not, with 46% (UCEC), 53% (COAD/READ), and 50% (SKCM) of the values for affected samples falling within the interquartile range (i.e., the middle 50%) of the distribution of their respective genes. These results indicate that the ZF mutations are affecting genes that are expressed at levels sufficient to play an active role in cancer (Fig 3).

### Mutations are concentrated in certain zinc finger genes

**Fig 4:**
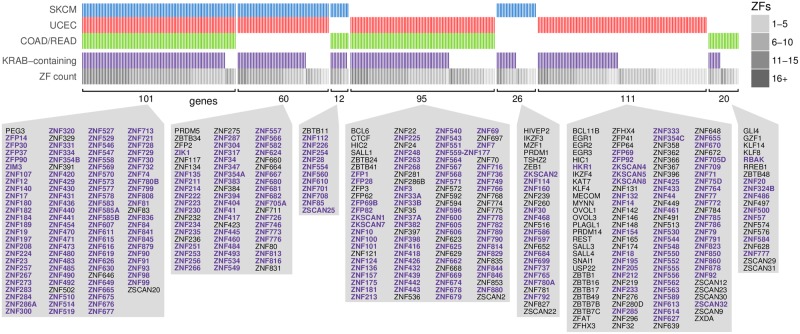
Zinc finger genes mutated in uterine cancer, colorectal cancer and melanoma overlap significantly. Each mutated gene is represented as a column in which a blue, red, or green bar indicates whether a ZF position 11 (SKCM) or position 9 (UCEC and COAD/READ) missense mutation was observed in that gene in any tumor. Also shown is whether each gene contains a KRAB domain, the number of ZF domains in the gene, and the names of the genes in each of the seven groups sorted alphabetically (purple bolded genes contain a KRAB domain).

We found 425 distinct ZF genes that had missense mutations in either p9 in COAD/READ or UCEC or in p11 in SKCM. The sets of these genes mutated in each cancer type show considerable overlap (Fig 4). Within each cancer type, these mutations often occur in the same gene, either in separate individuals or in the same individual, with the highest counts occurring in *ZNF721* in UCEC (17 mutations, 14 individuals) and COAD/READ (11 mutations, 8 individuals), and *ZNF208* in SKCM (32 mutations, 17 individuals). To test whether p9 and p11 missense mutations disproportionately affect some ZF genes, we calculated the distribution of the number of genes that would be mutated in p9 or p11 by randomizing the mutations within all p9 or p11 sites while preserving trinucleotide context; these permutation tests account for both the number of ZF domains each gene contains and the nucleotide contexts in these positions of the domains (see Methods). The actual mutations are concentrated in fewer genes than expected; they occur in 367 genes in UCEC, 228 genes in COAD/READ and 199 genes in SKCM, and these values are 16.4%, 18.0% and 24.4% lower than the average number observed in randomizations (all three empirical *p*-values < 0.0001). In other words, p9 and p11 mutations tend to be preferentially found in subsets of ZF genes.

### Mutations target KRAB zinc finger genes

Given that p9 and p11 missense mutations are distributed unevenly across the entire set of ZF genes, we next considered whether particular subsets of ZF genes were disproportionately affected. We observed that of the mutated ZF genes, 68% contain a KRAB repressor domain, whereas only 51% of all ZF genes included in our study contain a KRAB domain. This proportion increases substantially when considering the 101 genes found to be mutated across all three cancer types, as 94 (93%) of them contain a KRAB domain (Fig 4). Further, the actual fraction of missense mutations that occur at these positions in KRAB-containing genes is higher than expected by chance (empirical *p*-values ≤ 0.0001 for all three cancers using the p9 and p11 permutations described in the previous section). Thus, KRAB-containing genes, and presumably their shared biological roles, are preferentially targeted by p9 and p11 mutations.

### Biological processes and pathways of mutated genes are consistent with a role in cancers

ZF genes with p9 and p11 missense mutations are largely understudied, with Gene Ontology (GO) biological process (BP) annotations unrelated to transcriptional regulation associated with only 28% of the p9 or p11 mutated ZF genes. Accordingly, GO functional enrichment on the sets of ZF genes with p9 or p11 missense mutations in each cancer type yielded only general terms, largely related to transcription and regulation. Only 12 mutated ZF genes are associated with KEGG pathways; remarkably, however, all but one of these are associated either with cancer pathways or with signaling pathways regulating pluripotency of stem cells. Further, when analyzing the proteins that interact [32] with mutated ZF genes (see Methods), the most significantly enriched KEGG pathways of the partners were Notch signaling pathway (UCEC, *q* = 2.1e-13 and COAD/READ, *q* = 0.12), a well-known cancer pathway [33] and viral carcinogenesis (SKCM, *q* = 0.16). Many of the most highly enriched GO terms among the partners were related to transcription and chromatin organization, as would be expected for KRAB-containing ZF genes. Interestingly, keratinization was the most significantly enriched BP term among partners in COAD/READ and SKCM and the third-most enriched in UCEC, largely due to numerous ZF protein interactions with keratins and keratin-associated proteins.

Position 9 and 11 missense mutations are found in several known cancer genes. These include 11 genes—*BCL6*, *CTCF*, *PRDM1*, *BCL11B*, *KLF4*, *MECOM*, *SALL4*, *ZNF384*, *ZNF331*, *ZFHX3* and *ZBTB16*—which are included in the Cancer Gene Census (CGC) [34], a list of genes causally implicated in cancer. Additionally, there are several other p9 and p11 mutated ZF proteins that are not in the CGC but nevertheless have some support for a role in cancer. For example, *PEG3*, which had a p9 missense mutation in three UCEC tumors and one COAD/READ tumor and an H11Y mutation in one SKCM tumor, has been implicated in the tumor necrosis factor response pathway as part of a protein complex that activates NF*κ*B [35]; it also plays a critical role in p53-mediated apoptosis [36]. *ZNF382* had these mutations in six UCEC tumors and three COAD/READ tumors, and has been implicated as a tumor suppressor [37] that is silenced in multiple carcinoma cell lines, including colon, cervical and gastric. *ZNF420*, also known as *APAK*, had missense mutations at p9 in five UCEC tumors and one COAD/READ tumor, and at p11 in one SKCM tumor; this protein interacts with p53 and in normal cells suppresses p53-mediated apoptosis [38]. Further, other p9 and p11 mutated proteins are implicated in the p53 pathway, including ZNF273, ZNF677 and ZFP28, which are the three ZF proteins recently found to interact with p53 binding protein p53bp1 [17]. Three other genes with p9 or p11 missense mutations, *ZNF281*, *ZNF148* (*ZBP89*), and *ZEB1*, have also been identified as ZF genes important in cancer onset and progression [39]. Overall, many of the known biological processes and pathways of mutated ZF genes support their roles in cancer.

## Discussion

We have found that two positions within ZF domains are recurrently mutated in three cancer types. Our findings are robust in a variety of settings, including the use of different mutation callers (see S1 Text), and considering subsets of patients both with and without mutations within *POLE*. A previous analysis by Miller et al. [22] found that 82 positions within a diverse set of domains are hotspots that accumulate mutations more often than expected assuming that mutations are uniformly distributed across each domain. One of these identified hotspots is position 7 of Pfam domain family zf-H2C2_2; we determined that it corresponds to p9 in the present study. That hotspot was not investigated further, nor was a hotspot corresponding to p11 reported. In contrast, we demonstrate that the mutations affect two functionally important positions within ZFs, and occur more often than expected when taking into account context-specific, per-gene, and per-patient mutation rates. Further, we establish that the genes affected by these mutations tend to be more highly mutated than other genes, and are expressed at levels comparable to other cancer-relevant genes.

Several factors seem to contribute to the enrichment of mutations seen at specific ZF domain positions in UCEC, COAD/READ, and SKCM. These cancer types have relatively high mutation rates, and the positions that are affected in these cancers have nucleotide contexts that are consistent with their overall mutational signatures. Indeed, there can be disparities in both the expected and observed mutation counts at functionally similar positions; for example, the trinucleotide contexts of the first codon position at p7 (which corresponds to the other histidine coordinating zinc) are not especially prone to mutation in SKCM as they are for p11. Conversely, other positions with similar contexts as those affected do not accumulate mutations—including p8 in UCEC and COAD/READ (S2 Fig)—suggesting the importance of the specific positions affected. While there do not appear to be specific, highly mutated positions within ZF domains when analyzing other cancer types (panel B in S5 Fig), the enrichment of missense mutations in relation to synonymous mutations in the set of affected ZF genes holds true for other cancer types as well (panel C in S5 Fig), suggesting that these ZF genes may be broadly affected across many cancer types.

The two altered positions we uncover are critical to the intended biological function of ZF proteins, as mutations in p11 abrogate zinc coordination and thus domain stability [25], while mutations in p9 alter the positioning of ZF domains with respect to DNA and thus affect DNA-binding preferences [23]. Nevertheless, while p9 and p11 mutations alter or even destroy the ability of a particular ZF domain to bind DNA, because ZF proteins bind DNA via multiple domains, the protein may still be able to bind DNA albeit with different binding preferences. Thus, the overall impact at the protein level may result in both the loss and gain of regulatory targets.

The mutated ZF genes, as well as ZF genes in general, are understudied and the pathways and functions that they participate in are largely unknown. Furthermore, ZF genes that have been characterized have been shown to take part in a diverse set of pathways, including differentiation, development, growth and metabolism [40]. We thus expect that a wide spectrum of functions may be affected by the observed mutations; this is consistent with the fact that gene expression dysregulation is rampant in human cancers, and typically hundreds of genes are differentially expressed between normal and tumor samples [7]. For these reasons, it is difficult to uncover downstream expression changes due to these mutated ZF genes; further experimental work would be helpful in revealing such effects.

Despite the diversity of mutated genes, at least two broad but overlapping regulatory functions are prevalent among them. First, KRAB-containing ZF proteins, which are enriched in our set of mutated genes, play a significant role in shaping chromatin. In particular, KRAB proteins can recruit their co-factor KAP1/TRIM28, which then serves as a scaffold for bringing together factors that induce heterochromatin; this can have long-range effects, with repression evident tens of kilobases away [41]. Given that recent ChIP experiments have revealed that KRAB-containing ZF proteins can have up to 15,000 binding sites across the human genome [17, 18], the mutations we observe can have a significant effect on widespread epigenetic changes. Indeed, proteins affecting chromatin organization are increasingly being implicated in oncogenesis [19]. Second, many KRAB-containing ZF proteins have been implicated in repressing retroelements, as well as the genes neighboring these elements, both in embryonic stem cells and in fully differentiated cells [15, 16, 42]. Two recent large-scale ChIP-seq and ChIP-exo experiments on ZF proteins have determined genomic binding profiles for 231 of the 425 genes with p9 (UCEC, COAD/READ) or p11 (SKCM) missense mutations [17, 18], and the ChIP binding peaks of 156 of them were found to overlap significantly with specific retroelements in at least one of these two studies. Thus, misregulation of retroelements—or perhaps their nearby genes—may result from the p9 and p11 somatic mutations we observe. Intriguingly, transposable element expression and insertions have been observed in cancers and have been proposed to provide a selective advantage for tumorigenesis [20, 43].

In conclusion, somatic mutations at specific positions are pervasive within ZF genes, the largest class of human transcription factors, in at least three cancer types. We propose that these mutations are key contributors to widespread transcriptional deregulation in the tumors in which they are found. The frequency, distribution and enrichment of these mutations across ZF domains strongly suggest that they confer a selective growth advantage to cancer cells. The specific ZF genes mutated vary across tumors, however, and while certain shared functions are likely involved, discovering the full range of downstream effects of these shared yet distinct mutations is an exciting avenue for future research.

## Materials and methods

### Mutation data

Somatic mutations called by the MuTect2 software [44] were downloaded from the Genomic Data Commons portal. Point mutations from a total of 10,468 tumor samples were examined, spanning 32 cancer types after combining colon and rectal adenocarcinomas into a single cancer type (S1 Table). The TCGA data was processed as previously described [6], obtaining a set of reference gene transcripts and corresponding protein sequences onto which the mutations were mapped. If a gene had multiple isoforms, the one allowing the largest number of mutations to be mapped was retained. We also confirmed our analysis with mutations called by three other variant callers, and when run on a smaller set of stringently filtered samples (see S1 Text).

### Identifying mutations within zinc finger genes

The HMMER function hmmsearch (versions 2.3.2 and 3.0) was run on Ensembl human protein sequences using 12 Pfam HMM profiles from the Cys2-His2 ZF clan: PF00096, PF12756, PF13912, PF12171, PF13913, PF13909, PF12874, PF12907, PF02892, PF06220, PF09237, and PF11931. Matches to these profiles were further required to have an E-value less than 0.1 and to match *CX*_2_*CX*_9_Ψ*X*_2_*HX*_3_[*H*|*C*], where Ψ is a large, hydrophobic amino acid and the final amino acid can be H or C.

Mutations across individual ZF domains were aggregated according to the position in which they occurred within the domain. Mutations were only considered if the entire ZF domain exactly matched the protein sequence from RefSeq. These criteria only excluded a few mutations in each cancer type due to variations between Ensembl and RefSeq sequences.

### Context-based permutation tests

For each cancer type, we considered all ZF genes, and computed for each position within the ZF domain the sum of all missense mutations observed across the samples at that position (panel A in S5 Fig). We visually observed ZF p9 and p11 mutational peaks for UCEC, COAD/READ and SKCM. For these three cancer types, we assessed the significance of the total number of mutations observed in each position using trinucleotide-preserving randomizations, adapted from the method of Hodis et al. [30]. Specifically, observed point mutations (missense and silent) were shuffled within each ZF gene’s coding sequence such that each observed mutation can only be moved to another position that involves the same nucleotide, and has the same flanking nucleotides on either side. Once randomizations were performed for all genes, the numbers of mutations at each position were recomputed. This process was repeated 10,000 times, and for each position, the actual number of mutations was compared with the distribution of mutations arising from the permutations. Empirical *p*-values were obtained by calculating, for each position, the fraction of permutations where the total number of mutations were at least as high as the actual number.

We performed a related but different permutation analysis to determine whether mutations are concentrated within particular subsets of ZF genes. In particular, we repeatedly performed context-sensitive randomizations of the locations of p9 mutations in UCEC and COAD/READ samples across all p9 codons in the ZF genes considered in our analysis, and likewise for p11 mutations in SKCM samples across all p11 codons. To obtain empirical *p*-values, we counted the number of permutations that had fewer genes with missense mutations in p9 (likewise, p11) than in the unpermuted data, as well as the number of permutations that had a higher fraction of p9 (likewise, p11) missense mutations occurring in KRAB-containing ZF genes.

### Poisson binomial test for significance of mutations in specific contexts

We used the Poisson binomial distribution to determine whether the cumulative numbers of mutations affecting ZF p9 in COAD/READ and UCEC and p11 in SKCM were significantly higher than expected when taking into account per-patient context-dependent mutation rates. The parameters required to compute the Poisson binomial distribution are: (1) the number of trials, expressed as the total number of positions of interest (i.e., p9 or p11) across all ZF genes matching the context/s of interest (i.e., AGA in COAD/READ and UCEC and CCN/TCN in SKCM, on either strand); and (2) the per-individual mutation rates for the context/s of interest, computed across the whole exome. The poibin R package [45] was used to compute the probability of observing a number of mutations in the position of interest greater than or equal to the observed value.

### Missense and synonymous mutation rates

For each cancer type, we computed the per-gene missense mutation rate as the total number of missense mutations observed in a gene, divided by the number of nonsynonymous sites in the gene, and likewise with synonymous mutations and sites. The nonsynonymous/synonymous sites are proportional to the outcomes of the possible mutations per position, and together sum to the length of the coding sequence.

### Functional analysis

Gene Ontology (GO) and KEGG functional enrichments on mutated gene sets were determined using the hypergeometric distribution, with the background set of all annotated genes, and with *q*-value correction.

To test enrichment of annotation sets in the interaction partners of the mutated ZF genes, we used the undirected form of the NEAT method [46], which is based on the hypergeometric distribution and tests for the enrichment of interactions between two gene sets. We performed the test between each set of ZF genes in question and each annotation set, with the modification that we computed one-sided *p*-values using hypergeometric tests and then computed *q*-values [47] across ontology terms or pathways within each cancer type.

## Supporting information

S1 Text Mutation peaks at p9 and p11 are robust to mutation calls. (PDF)

S1 Fig Zinc finger domains with R9I mutations are within arrays of domains more often than expected. Four sets of ZF domains are compared: all ZF domains in the human genome, those with any missense mutation in any sample from the three cancer types, those that contain a position 11 histidine to tyrosine substitution at least once in any of the three cancer types, and those that contain at least one position 9 arginine to isoleucine substitution. The fraction of ZF domains in each set whose C-terminal boundary is at most 12 amino acids from a subsequent zinc finger domain is shown, with *p*-values obtained from binomial tests using the larger set in the comparison as the expected fraction and the subset as the trial group.
(PDF)

S2 Fig Trinucleotide-preserving randomizations across Cys2His2 zinc finger genes. Bars give the actual missense mutation counts for each of 21 positions in a classic Cys2His2 zinc finger domain. Data over 5483 domains in 642 genes. Boxplots show the distributions of counts after observed mutations are shuffled 10,000 times across each gene in a trinucleotide context-preserving manner.
(PDF)

S3 Fig Confirmation of ZF mutation patterns. **(a)** To check whether the mutation peaks were robust with respect to the choice of mutation caller, we repeated our domain analysis with the three other versions of each mutation dataset, as processed by the MuSE, SomaticSniper, and VarScan2 callers. **(b)** We repeated our domain analysis with a filtered version of mutation calls (see S1 Text). The number of samples in the filtered dataset was 247 for UCEC, 224 for COAD/READ, and 253 for SKCM. **(c)** Position 9 and 11 peaks remain when only mutations at genomic locations with the highest possible 36-mer mappability scores are considered. **(d)** Position 9 peaks remain when samples with excessive sequencing damage are removed. **(e)** Even when all mutations in ZF domains that had at least one R9I mutation in that cancer type are excluded, more missense mutations occur at position 9 than at other positions.
(PDF)

S4 Fig Allele frequency of R9I and H11Y mutations. For each cancer type, the tumor sample allele frequencies of R9I or H11Y mutations are compared with those of all other exome mutations involving the same base substitution, as well as those of all exome mutations involving other base substitutions.
(PDF)

S5 Fig Summary of ZF mutations across all cancer types. **(a)** Missense mutations in ZF domains are shown for the nine cancer types in which they occur most frequently, as well as for the remaining 23 cancer types in aggregate. **(b)** Missense mutation counts from panel A normalized by number of samples per cancer type (or total number of samples across cancer types in the Other group). Colors correspond to the groups in panel A. **(c)** For each cancer type, normalized nonsynonymous to synonymous mutation ratios (as calculated for Fig 2d) per gene are grouped by whether the gene is in the combined set of genes with a p9 (UCEC, COAD/READ) or p11 (SKCM) missense mutation. Mutations in the 23 remaining cancer types were aggregated into a single group for calculating per-gene ratios.
(PDF)

S1 Table All cancer types examined, with abbreviations and sample counts. (PDF)

S1 Dataset List of ZF genes with p9 and p11 missense mutations. For each gene, the number of missense mutations at p9 or p11 in each cancer type is given. Also shown is whether the gene has a KRAB domain, and the number of ZF domains in the gene that were considered in this study.
(TXT)
