# Supplementary material for: Two critical positions in zinc finger domains are heavily mutated in three human cancer types
Source: PLoS Comput Biol. 2018 Jun 28;14(6):e1006290. doi: 10.1371/journal.pcbi.1006290 (PMC6040777; doi:10.1371/journal.pcbi.1006290)
Supplement: S1 Text — (PDF) [file pcbi.1006290.s001.pdf]

Supplementary Text

for "Two critical positions in zinc finger domains are heavily mutated in three human cancer types"

Mutation peaks at p9 and p11 are robust to mutation calls. In the main text, we analysed mutations called by MuTect2 [1]. However, the mutational peaks are also evident when using mutations called by MuSE [2], SomaticSniper [3] and VarScan2 [4] (Supplemental Figure S3a), as well as when using a filtered dataset [5] that began with mutation data from various callers for a subset of the samples that were available in 2013 and then removed known false positives and germ line variations found in dbSNP [6] (Supplemental Figure S3b). To confirm that the peaks were not a result of misaligned reads, we examined the mappability scores of the genomic locations of the mutations (ENCODE Accession ENCSR821KQV [7]). We found that the peaks remained when conservatively filtering mutations by requiring perfect 36-mer mappability scores (Supplemental Figure S3c).

Recently, it has been reported that in some sequencing samples, many low to moderate frequency (1 to 5%) G→T variant calls are false positives caused by DNA damage incurred during sample preparation [8]. To rule out the possibility that the AGA→ATA mutations (R9I) found in UCEC and COAD/READ were found in samples containing a large number of experimental artifacts, we used the Damage-estimator code provided by Chen et al. [8] in their study. Only 4 of 55 UCEC and 5 of 15 COAD/READ samples with R9I mutations were characterized as damaged via this software. Moreover, the ZF position 9 mutation peaks persist even when these samples are removed (Supplemental Figure S3d), and even when all domains with AGA→ATA mutations at position 9 are removed (Supplemental Figure S3e). Finally, box plots of the tumor sample allele frequencies of R9I UCEC and COAD/READ mutations and H11Y SKCM mutations are given in Supplemental Figure S4, and demonstrate that these mutations are not low frequency, but with median frequencies of 33%, 29% and 31%, respectively.

References

1. DePristo, M. A. et al. A framework for variation discovery and genotyping using next-generation DNA sequencing data. *Nature Genetics* 43, 491498 (2011).

2. Fan, Y. et al. MuSE: accounting for tumor heterogeneity using a sample-specific error model improves sensitivity and specificity in mutation calling from sequencing data. *Genome Biology* 17, 178 (2016).

3. Larson, D. E. *et al.* SomaticSniper: identification of somatic point mutations in whole genome sequencing data. *Bioinformatics* 28, 311–317 (2011).

4. Koboldt, D. C. *et al.* VarScan 2: somatic mutation and copy number alteration discovery in cancer by exome sequencing. *Genome Research* 22, 568576 (2012).

5. Kandoth, C. *et al.* Mutational landscape and significance across 12 major cancer types. *Nature* 502, 3339 (2013).

6. Sherry, S. T. *et al.* dbSNP: the NCBI database of genetic variation. *Nucleic Acids Res* 29, 308311 (2001).

7. ENCODE Project Consortium. An integrated encyclopedia of DNA elements in the human genome. *Nature* 489, 57-74 (2012).

8. Chen, L., *et al.* DNA damage is a pervasive cause of sequencing errors, directly confounding variant identification. *Science* 355, 752756 (2017).
