# Supplementary figures and images for: Two critical positions in zinc finger domains are heavily mutated in three human cancer types
Source: PLoS Comput Biol. 2018 Jun 28;14(6):e1006290. doi: 10.1371/journal.pcbi.1006290 (PMC6040777; doi:10.1371/journal.pcbi.1006290)

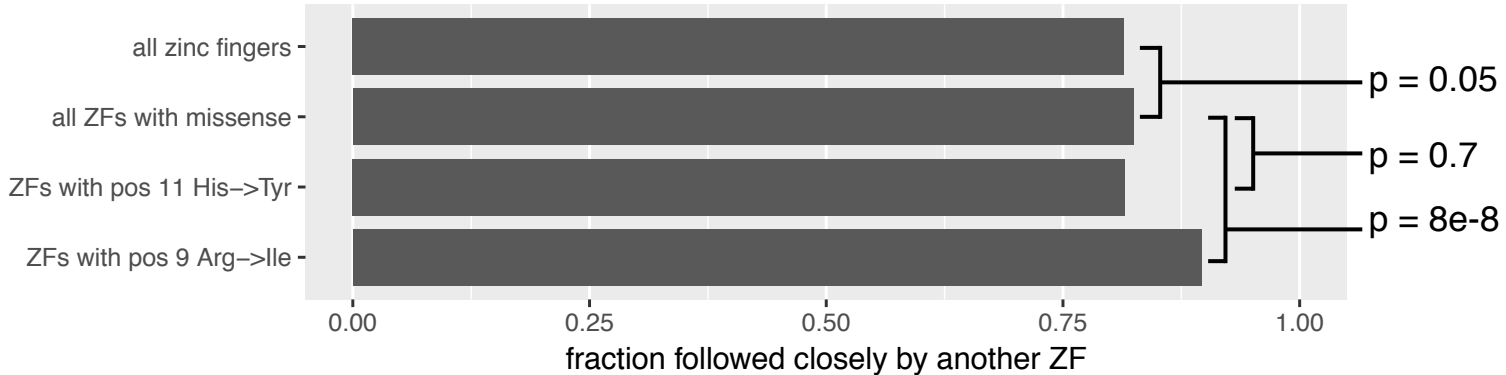

Supplement: S1 Fig — Four sets of ZF domains are compared: all ZF domains in the human genome, those with any missense mutation in any sample from the three cancer types, those that contain a position 11 histidine to tyrosine substitution at least once in any of the three cancer types, and those that contain at least one position 9 arginine to isoleucine substitution. The fraction of ZF domains in each set whose C-terminal boundary is at most 12 amino acids from a subsequent zinc finger domain is shown, with p-values obtained from binomial tests using the larger set in the comparison as the expected fraction and the subset as the trial group. (PDF) [file pcbi.1006290.s002.pdf]

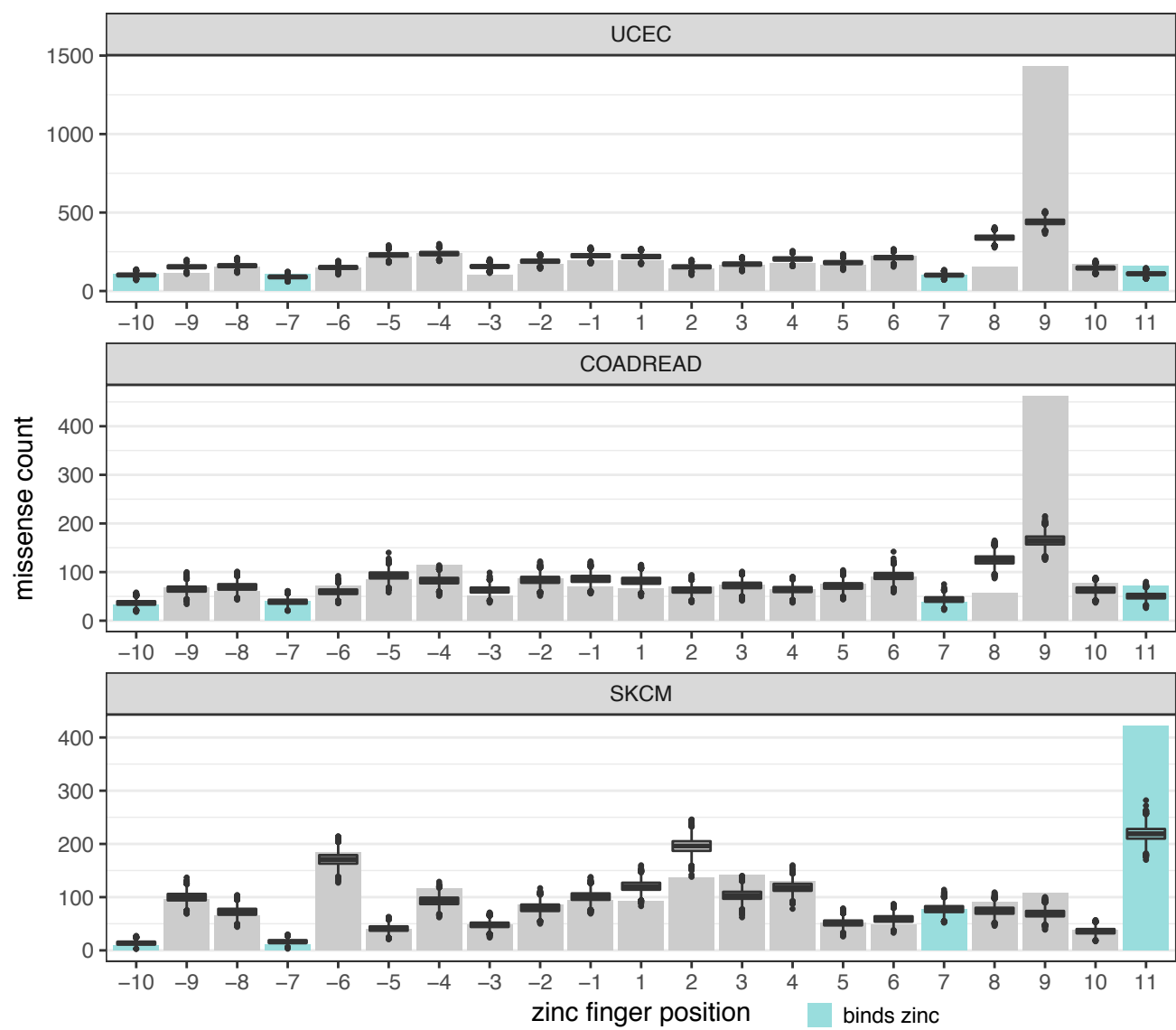

Supplement: S2 Fig — Bars give the actual missense mutation counts for each of 21 positions in a classic Cys2His2 zinc finger domain. Data over 5483 domains in 642 genes. Boxplots show the distributions of counts after observed mutations are shuffled 10,000 times across each gene in a trinucleotide context-preserving manner. (PDF) [file pcbi.1006290.s003.pdf]

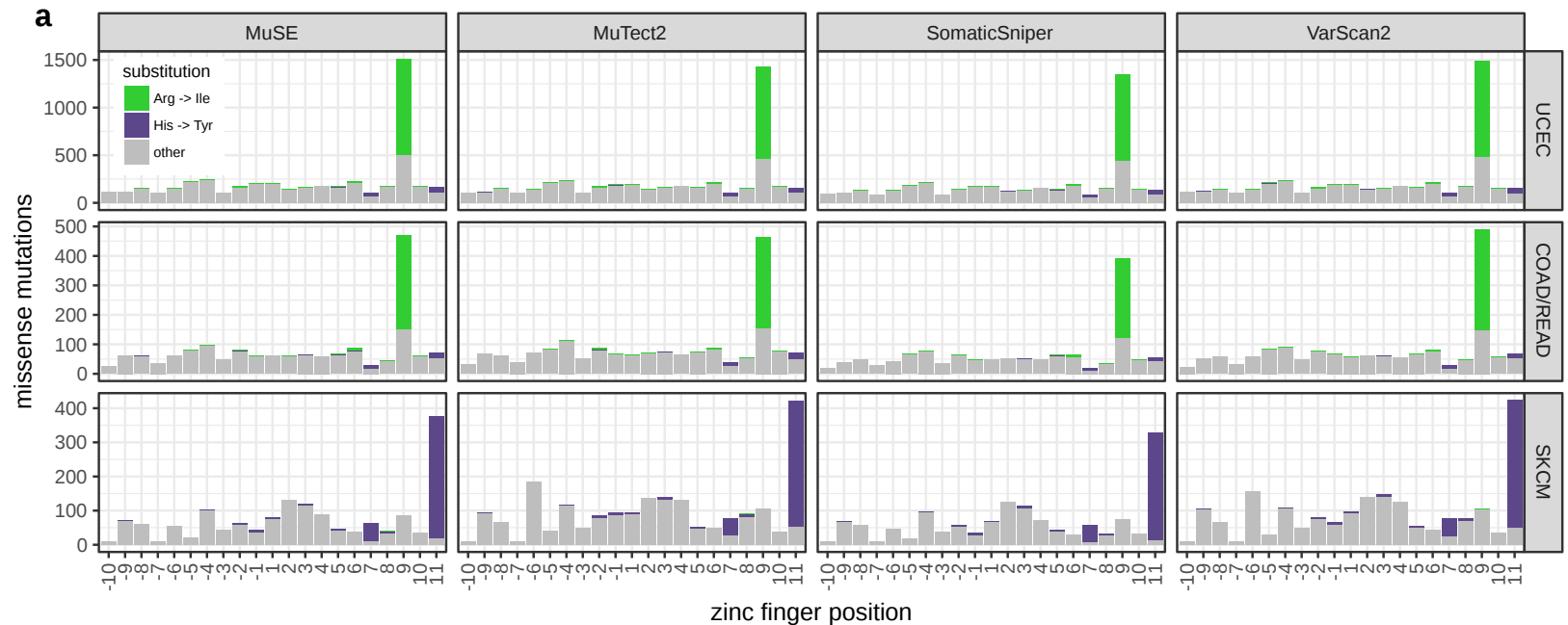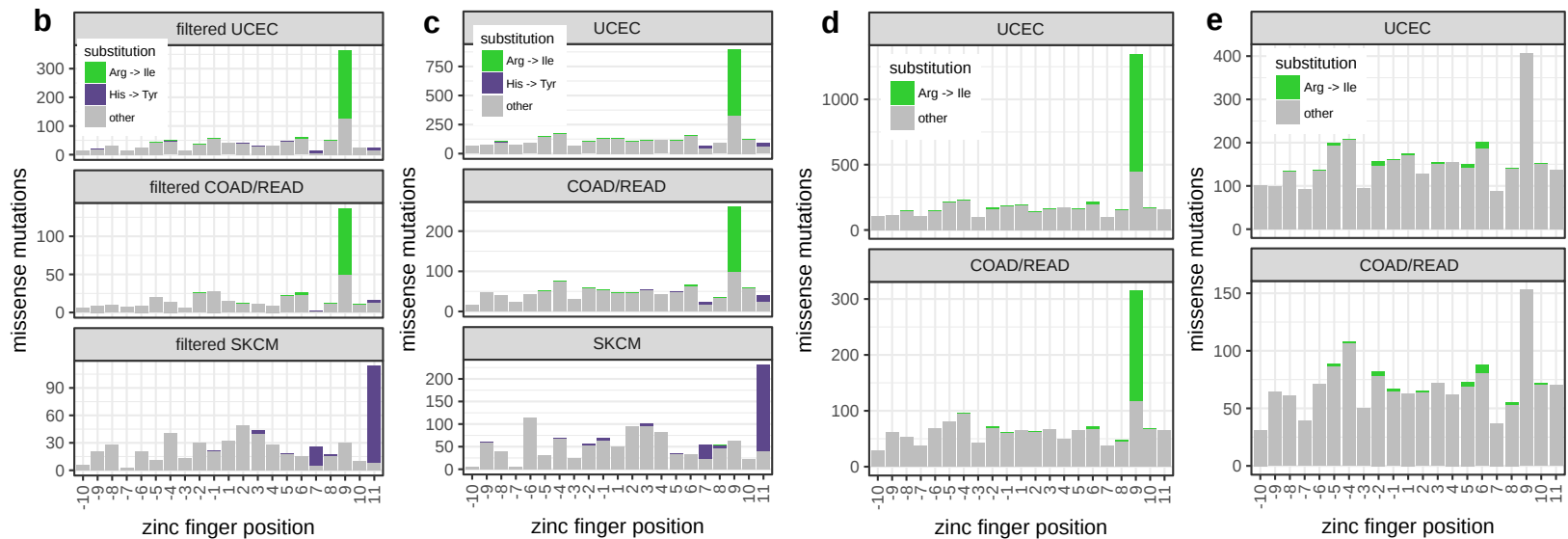

Supplement: S3 Fig — (a) To check whether the mutation peaks were robust with respect to the choice of mutation caller, we repeated our domain analysis with the three other versions of each mutation dataset, as processed by the MuSE, SomaticSniper, and VarScan2 callers. (b) We repeated our domain analysis with a filtered version of mutation calls (see S1 Text). The number of samples in the filtered dataset was 247 for UCEC, 224 for COAD/READ, and 253 for SKCM. (c) Position 9 and 11 peaks remain when only mutations at genomic locations with the highest possible 36-mer mappability scores are considered. (d) Position 9 peaks remain when samples with excessive sequencing damage are removed. (e) Even when all mutations in ZF domains that had at least one R9I mutation in that cancer type are excluded, more missense mutations occur at position 9 than at other positions. (PDF) [file pcbi.1006290.s004.pdf]

Mutation type

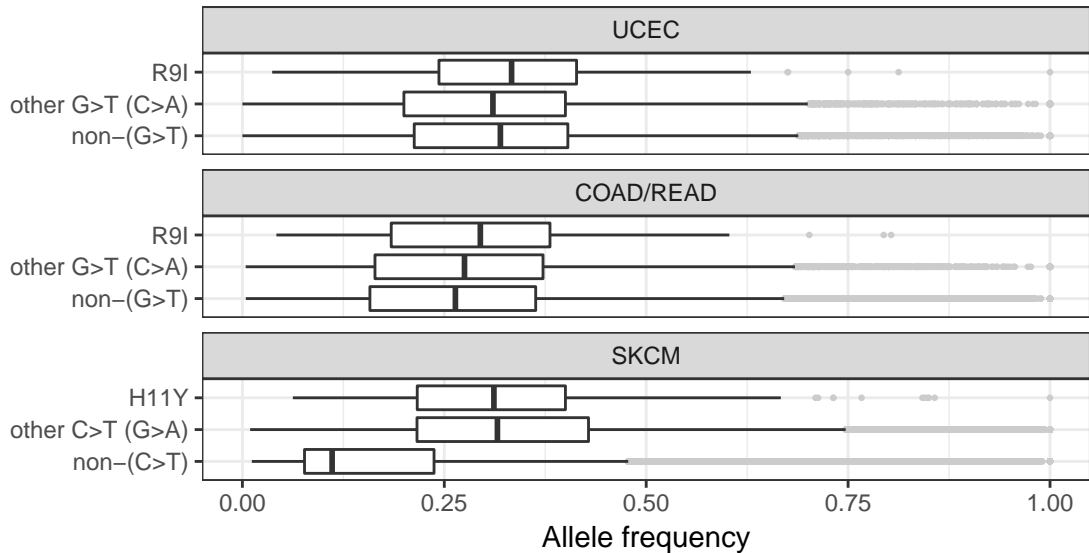

Supplement: S4 Fig — For each cancer type, the tumor sample allele frequencies of R9I or H11Y mutations are compared with those of all other exome mutations involving the same base substitution, as well as those of all exome mutations involving other base substitutions. (PDF) [file pcbi.1006290.s005.pdf]

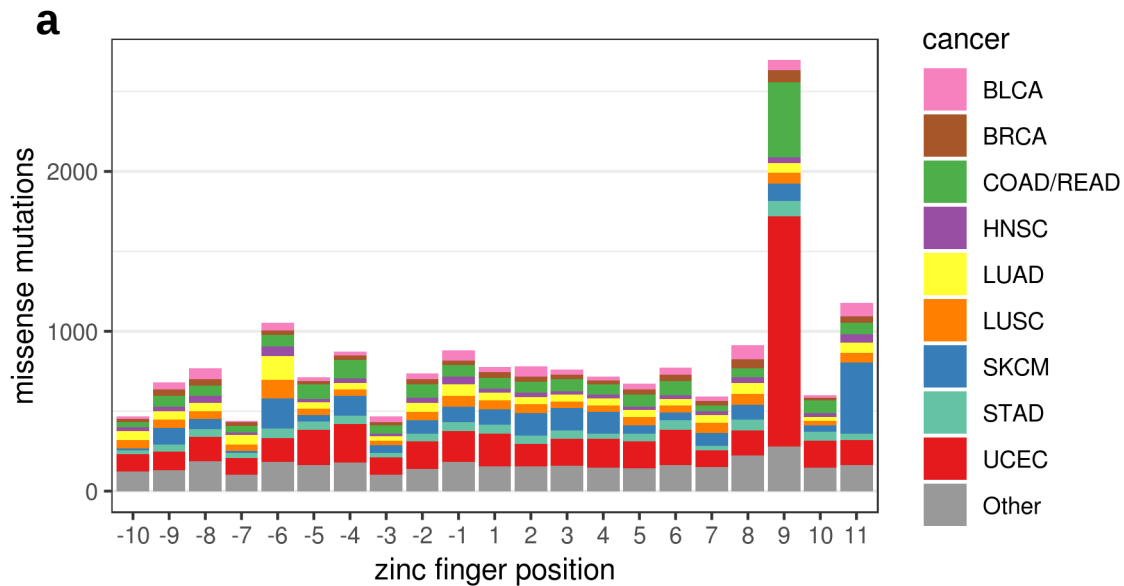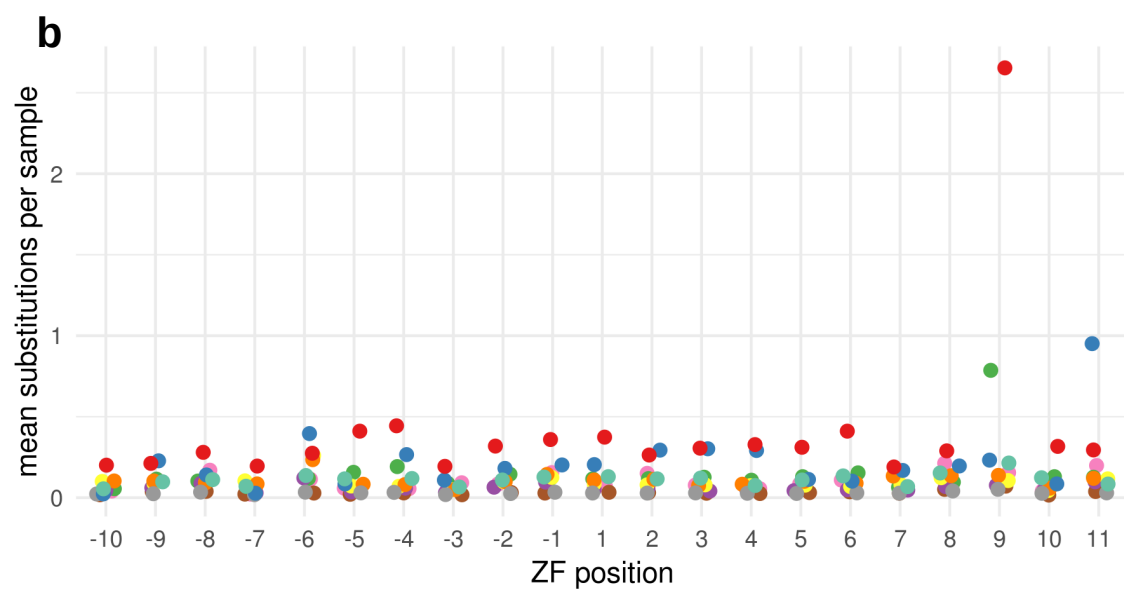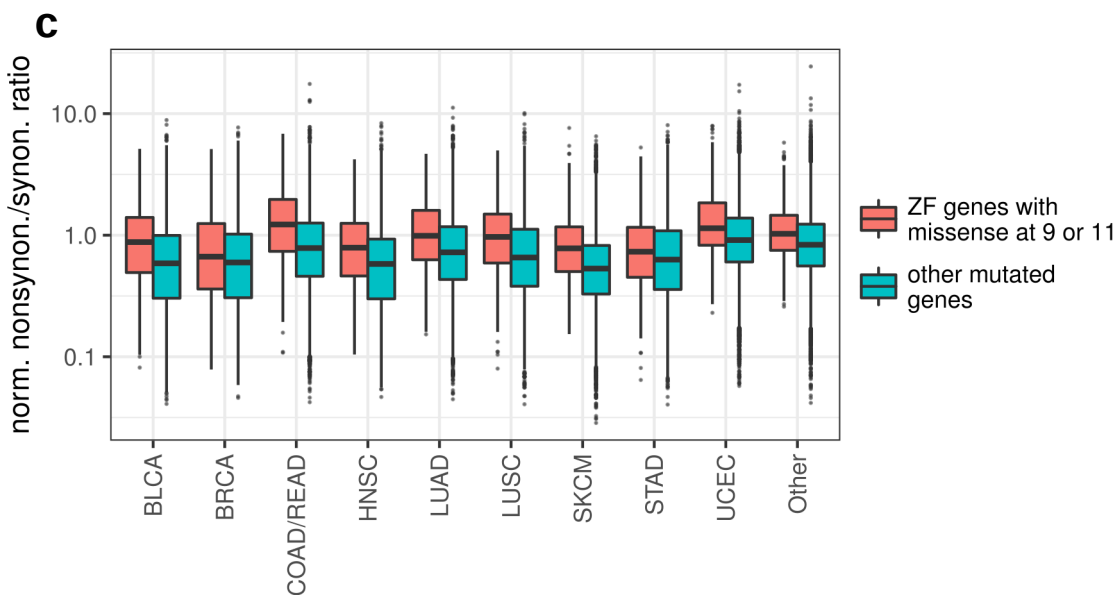

Supplement: S5 Fig — (a) Missense mutations in ZF domains are shown for the nine cancer types in which they occur most frequently, as well as for the remaining 23 cancer types in aggregate. (b) Missense mutation counts from panel A normalized by number of samples per cancer type (or total number of samples across cancer types in the Other group). Colors correspond to the groups in panel A. (c) For each cancer type, normalized nonsynonymous to synonymous mutation ratios (as calculated for Fig 2d) per gene are grouped by whether the gene is in the combined set of genes with a p9 (UCEC, COAD/READ) or p11 (SKCM) missense mutation. Mutations in the 23 remaining cancer types were aggregated into a single group for calculating per-gene ratios. (PDF) [file pcbi.1006290.s006.pdf]
