# Supplementary material for: Two critical positions in zinc finger domains are heavily mutated in three human cancer types
Source: PLoS Comput Biol. 2018 Jun 28;14(6):e1006290. doi: 10.1371/journal.pcbi.1006290 (PMC6040777; doi:10.1371/journal.pcbi.1006290)
Supplement: S1 Table — (PDF) [file pcbi.1006290.s007.pdf]

Supplementary Table

for "Two critical positions in zinc finger domains are heavily mutated in three human cancer types"

Table S1: **All cancer types examined, with abbreviations and sample counts.**

**Symbol**	**Name**	**Samples**
ACC	Adrenocortical Carcinoma	92
BLCA	Bladder Urothelial Carcinoma	413
BRCA	Breast Invasive Carcinoma	1050
CESC	Cervical Squamous Cell Carcinoma and Endocervical Adenocarcinoma	307
CHOL	Cholangiocarcinoma	51
COAD/READ	Colorectal Adenocarcinoma	594
DLBC	Lymphoid Neoplasm Diffuse Large B-cell Lymphoma	48
ESCA	Esophageal Carcinoma	185
GBM	Glioblastoma Multiforme	408
HNSC	Head and Neck Squamous Cell Carcinoma	510
KICH	Kidney Chromophobe	66
KIRC	Kidney Renal Clear Cell Carcinoma	340
KIRP	Kidney Renal Papillary Cell Carcinoma	289
LAML	Acute Myeloid Leukemia	140
LGG	Brain Lower Grade Glioma	525
LIHC	Liver Hepatocellular Carcinoma	377
LUAD	Lung Adenocarcinoma	568
LUSC	Lung Squamous Cell Carcinoma	494
MESO	Mesothelioma	83
OV	Ovarian Serous Cystadenocarcinoma	442
PAAD	Pancreatic Adenocarcinoma	180
PCPG	Pheochromocytoma and Paraganglioma	182
PRAD	Prostate Adenocarcinoma	497
SARC	Sarcoma	259
SKCM	Skin Cutaneous Melanoma	470
STAD	Stomach Adenocarcinoma	439
TGCT	Testicular Germ Cell Tumors	154
THCA	Thyroid Carcinoma	502
THYM	Thymoma	123
UCEC	Uterine Corpus Endometrial Carcinoma	543
UCS	Uterine Carcinosarcoma	57
UVM	Uveal Melanoma	80
